# Overexpression of soybean *GmDHN9* gene enhances drought resistance of transgenic *Arabidopsis*

**DOI:** 10.1080/21645698.2024.2327116

**Published:** 2024-04-02

**Authors:** Jiayi Fan, Yuzhe Zhang, Hongji Sun, Ruijie Duan, Yushi Jiang, Xinyu Wang, Yao Sun, Zhipeng Luo, Peiwu Wang, Shuyan Guan, Siyan Liu, Xuhong Fan, Peng Jiao, Yunpeng Wang, Jinhui Yang, Zunyue Zhang, Huiwei Yu

**Affiliations:** a College of Agronomy, Jilin Agricultural University, Changchun, China; b College of Life Sciences, Jilin Agricultural University, Changchun, China; c Changchun Vocational and Technical College, Changchun Vocational Institute of Technology, changchun, China; d Joint International Research Laboratory of Modern Agricultural Technology, Ministry of Education, Jilin Agricultural University, Changchun, China; e Soybean Research Institute, Jilin Academy of Agricultural Sciences, Changchun, China; f Daan Branch of Baicheng City Tobacco company of Jilin Province, Baicheng, China

**Keywords:** dehydrin, drought resistance, function, gene expression, reactive oxygen species

## Abstract

Soybean is one of the important oil crops and a major source of protein and lipids. Drought can cause severe soybean yields. Dehydrin protein (DHN) is a subfamily of LEA proteins that play an important role in plant responses to abiotic stresses. In this study, the soybean *GmDHN9* gene was cloned and induced under a variety of abiotic stresses. Results showed that the *GmDHN9* gene response was more pronounced under drought induction. Subcellular localization results indicated that the protein was localized in the cytoplasm. The role of transgenic Arabidopsis plants in drought stress response was further studied. Under drought stress, the germination rate, root length, chlorophyll, proline, relative water content, and antioxidant enzyme content of transgenic Arabidopsis thaliana transgenic genes were higher than those of wild-type plants, and transgenic plants contained less O_2_^−^, H_2_O_2_ and MDA contents. In short, the *GmDHN9* gene can regulate the homeostasis of ROS and enhance the drought resistance of plants.

## Introduction

Soybean (*Glycine max* L.) can be grown under a wide range of cultivation conditions and has a rich nutritional value. It can be utilized to produce different soy products, soybean oil, okara, and feed.^1^ Abiotic stress caused by drought, a widespread meteorological hazard, is considered the most important limiting factor for soybean yields.^2^ The aerobic metabolism of plants that maintain life activities produces reactive oxygen species (ROS), and under normal circumstances, their production and elimination are in a state of dynamic equilibrium.

However, when plants are subjected to drought stress, the balance of reactive oxygen species (ROS) in the plant is disrupted and the ROS scavenging capacity is reduced, leading to the accumulation of ROS, resulting in cell damage and death.^3–5^

Elevated ROS levels lead to loss of organelle function, decreased metabolic function, lipid peroxidation, programmed cell death, and electrolyte leakage.^6^ At the same time, the plant’s ability to scavenge reactive oxygen species reflects, to a certain extent, the plant’s tolerance to abiotic stresses. In order to balance the level of reactive oxygen species in plants, plants themselves reduce the level of reactive oxygen species through a variety of antioxidant enzyme reactions. Superoxide dismutase (SOD), catalase (CAT), and peroxidase (POD) can convert reactive oxygen radicals (O_2_^−^) and H_2_O_2_ into H_2_O and O_2_, which in turn functions to scavenge reactive oxygen species (ROS).^7^ Chen et al. reported that the overexpression of *ZmNAC2* in maize enhanced the scavenging ability of ROS and enhanced the osmotic stress tolerance of transgenic *Arabidopsis*.^8^ Yan et al. found that *GhCDPK60* positively regulated the drought resistance of transgenic *Arabidopsis thaliana* and cotton by regulating the content of proline (Pro) and ROS levels.^9^ In another study, Wang et al. showed that *OsLEA1a* overexpression enhanced tolerance to different abiotic stresses in transgenic rice by inhibiting cell membrane damage and improving ROS clearance ability.^10^

Late embryonic developmental abundant proteins (LEAs) are a widespread class of proteins in plants that protect plant cells from abiotic stress damage. LEA proteins are categorized into eight types based on their conserved motifs, amino acid sequences, and phylogenetic relationships, namely LEA-1, LEA-2, LEA-3, LEA-4, LEA-5, LEA-6, SMP, and DHN. Yu et al.^11^ showed that overexpression of the rice *OsEm1* gene increased the sensitivity of rice to ABA and improved the plant’s osmotic coordination. Huang et al.^12^ found that the *OsLEA5* gene is involved in ABA-mediated antioxidant defense and plays a vital role in rice’s drought and salt stress response. Sun et al.^13^ suggested that *GsPM30* overexpression enhances salt and drought tolerance in Arabidopsis seedlings and at the seedling stage. Dehydrin (DHN), a thermostable protein belonging to a subfamily of the LEA protein family, is expressed in large quantities in the later stages of embryonic development and has a highly hydrophilic sequence.^14^ Dehydrogenases (DHNs) are proteins that are highly hydrophilic as well as stress-responsive to stress. Drought stress induced by drought causes dehydrogenase to accumulate during late embryonic development.^15^ DHN proteins have a wide range of molecular weights, ranging from 9 to 200 kDa. The lysine-rich repeat structural domain (EKKGIMDKIKEKLPG) is the only conserved motif in DHN and may play a key role in protein-lipid interactions.^16–19^ DHNs also contain multiple other conserved motifs, such as a tyrosine-rich Y fragment motif located at the N-terminal end of the protein-coding region (T/VDEYGNP) and an S segment containing a segment of 4–10 serine residues located between the Y and K segments.^20^ Protein conformation and ion-binding activity can be regulated by phosphorylation of the modified serine residue composition.^21^ There is a pair of hydrophobic Phe residues at the core of the F-segment sequence. The α segment is poorly conserved, mainly containing glycine and polar amino acids, which can interact with small molecular polar substances to improve the hydrophilicity of DHN and reduce the damage to proteins.^22^ Depending on the presence, deletion, and number of repetitions of these fragments, different combinations of structural domains can be formed,^23^ resulting in different taxa, classifying into six subclasses, namely: YnSKn, FnSKn, YnKn, SKn, Kn, and KnS.^24^

DHN resists stress such as drought by protecting intracellular protein activity, binding metal ions to scavenge intracellular reactive oxygen radicals, binding DNA, stabilizing cell membranes with phospholipids, protecting enzyme activity, and scavenging ROS.^25^ The *CaDHN3* gene showed enhanced tolerance to salt and drought stress by reducing the accumulation of ROS.^26^ Sorghum *SbDHN1* gene improved the protective effect under high temperature and osmotic stress conditions.^27^ Overexpression of polar *DHN* gene *PtrDHN-3* enhanced *A. thaliana*‘s tolerance to salt stress by increasing antioxidant enzyme activity.^28^ Tandem *TaDHN* genes responded strongly to stresses such as drought, cold, and high salt, whereas non-tandem genes responded poorly to all stress conditions. According to interaction network analysis, multiple DHN protein interactions are essential for plant resistance to abiotic stresses.^29^ Plant *DHNs* are widely involved in physiological processes in response to abiotic stress and play an important role in improving plant stress tolerance. Many *DHNs* have been identified in previous studies, but the function of *GmDHN9* has not been explored yet. In this study, we analyzed the response of the *GmDHN9* gene to drought, and the results suggested that *GmDHN9* might have a positive effect on the drought tolerance of plants by regulating ROS homeostasis.

### Experiments and Methods

#### Plant Material

In this study, soybean “JN28” and Colombian wild-type Arabidopsis thaliana col were planted in soil and placed in an artificial culture chamber (25°C, 16 h light/8 h dark) for cultivation. When soybean grew to the three-leaf stage, it was subjected to drought (10% PEG6000), salt (100 mM NaCl), ABA (100 μM ABA), and low-temperature (4°C) stress treatments, respectively, and the leaves were harvested at treatments of 0, 3, 6, and 12 h. Harvested samples were frozen in a freezer at −80°C for use in the subsequent experiments.

### Sequence Analysis

The *GmDHN9* gene (Gene ID:547842) cDNA sequence was derived from NCBI (https://www.ncbi.nlm.nih.gov/). The upstream 2000bp promoter analysis of the *GmDHN9* gene was performed using Plant CARE (http://bioinformatics.psb.ugent.be/webtools/plantcare/html/).

### RNA Extraction and qRT-PCR Analysis

When soybeans grew to the three-leaf stage, they were treated with 100 μM ABA, 10% PEG6000, 100 mM NaCl, and a low temperature of 4°C for 0, 3, 6, and 12 h. Next, their leaves were plucked, respectively, and RNA was extracted using the TRIzol method. The extracted RNA was then reverse transcribed into cDNA for qRT-PCR (GeneCopeia Co., Ltd.) detection and analyzed using the 2^−ΔΔCt^ method with three biological replicates.

### Subcellular Localization

The gene cloning vector was detected by PCR using specific primers to amplify the CDS sequence of the *GmDHN9* gene without an stop codon. Next, this was ligated into the pCAMBIA1302-GFP vector at the *Nco*I and *Spe*I sites to construct the pCAMBIA1302-GmDHN9-GFP recombinant vector. The recombinant vector was transferred into the Agrobacterium tumefaciens EHA105 strain, injected into tobacco epidermal cells, placed in a dark room at 25°C for 12 h, and then restored to light conditions for 48 h culturing, and observed using laser confocal microscopy.

### *Acquisition and Detection of Transgenic* Arabidopsis

An overexpression pCAMBIA3301-GmDHN9 vector was constructed, and the recombinant plasmid was transferred into the Agrobacterium strain EHA105 and transformed into wild-type A. thaliana by flower immersion. After screening and PCR detection by Basta, T_1_ transgenic A. thaliana-positive plants were obtained and propagated to T_3_ generation A. thaliana plants. Quantitative PCR detection of transgenic A. thaliana with T_3_ generation was performed, and three transgenic lines with relatively high expression were selected for drought resistance identification.

### Determination of Germination Rate and Root Length of Transgenic A. thaliana

The seeds of wild-type and T_2_ generation transgenic A. thaliana were sterilized with 75% alcohol for 1 min and 1% NaClO for 10 min, followed by rinsing with sterile water (3–4 times). Next, they were inoculated in 1/2 MS and 1/2 MS +100 mmol/L, 1/2 MS +200 mmol/L, and 1/2 MS +300 mmol/L mannitol, and maintained in dark conditions at 4°C for 3 days. They were then cultured in light conditions in the culture chamber, and the germination rate of A. thaliana was counted after 10 days. At the same time, A. thaliana was also cultured vertically to count their root length. Wild-type and T_2_ generation transgenic A. thaliana were planted in soil, and natural drought treatment was carried out in an artificial climate chamber, followed by rehydration process after 25 days of drought. Reference to ^3,30^. The wilting and recovery of A. thaliana were statistically observed.

### *Determination of Physiological and Biochemical Indexes of Transgenic* Arabidopsis

Wild-type and transgenic lines of A. thaliana were subjected to drought stress for 12 h (0 h as control), and the ROS content was determined in vivo by histochemical staining using 3,3’-diaminobenzidine (DAB) and p-nitroblue tetrachloride (NBT), which was based on the Soni ‘s method.^31^ The H_2_O_2_, O_2_^−^, malondialdehyde (MDA), chlorophyll, and Pro contents were determined using the methods described by Jiao et al..^4^ The determination of antioxidant enzyme activity, including SOD, POD, and CAT activities, was done according to Xiong’s methods.^32^

### Expression of Genes Associated with Drought Stress

Arabidopsis thaliana leaves with normal growth and under drought stress were selected. Total RNA was extracted, and the first strand of cDNA (Kangwei Reagent Technology Co., Ltd.) was synthesized for qRT-PCR (GeneCopeia Co., Ltd.) detection. The marker gene-specific primers DREB2A-F/R, RD17-F/R, RD26-F/R, and CBF3-F/R were used for qRT-PCR analysis, and AtEF1-F/R was used as the internal reference gene to calculate the expression of four stress-related marker genes.

### Statistical Analysis

Differences between the data were determined by analysis of one-way variance using SPSS (SPSS Inc., Chicago, IL, USA) and considered statistically significant at *p* < .05 (*) or *p* < .01 (**).

## Results

### GmDhn9 *Gene Was Significantly Upregulated Under Drought Stress*

**Figure 1.**
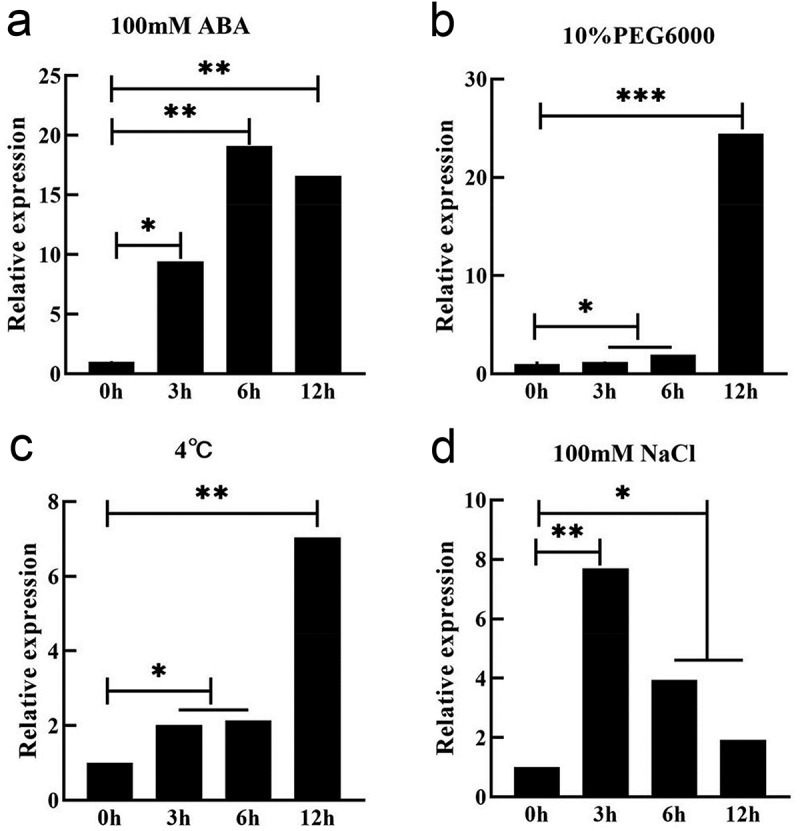
Expression of GmDHN9 gene under abiotic stress. (a) 100 μM ABA; (b) 10% PEG6000; (c) 4°C; (d) 100 mM NaCl. * *p* < .05; ** *p* < .01. All bars represent means ±SD, (*n* ≥ 3).

The conserved domain analysis of the gene (Fig. S1) showed that it contained the conserved domain of the DHN family. The gene encoded a protein of 226 amino acids, and the promoter sequence analysis of 2000bp upstream of the gene (Table S1) showed that it contained core promoter elements in response to drought, dehydration, abscisic acid, ABRE, and other related components, indicating that *GmDHN9* gene might respond to drought stress. Subsequently, we analyzed the expression of *GmDHN9* gene under drought (10% PEG6000), salt (100 mM NaCl), ABA (100 μM ABA), and low-temperature (4°C) stresses in soybean leaves, and the results showed (Fig. 1) that the expression of the *GmDHN9* gene was up-regulated under the different abiotic stresses, but was more pronounced in response to drought stress.

### Subcellular Localization

**Figure 2.**
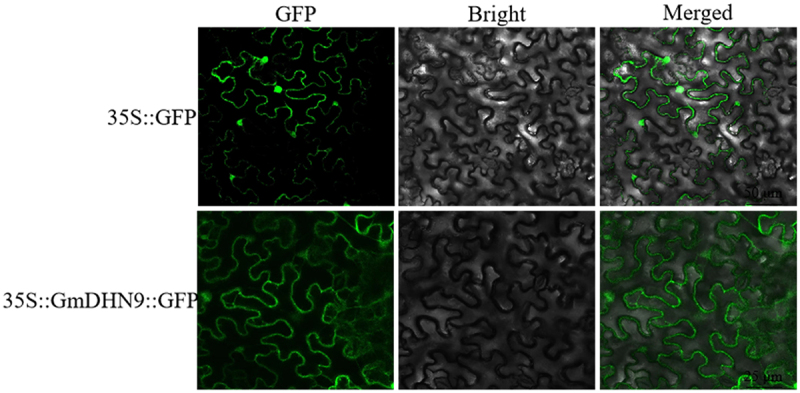
Subcellular localization analysis of GmDHN9 proteins in tobacco cells. （Bars = 25 μm).

Knowing the location of gene expression products is important for functional analysis of genes. To determine subcellular localization of *GmDHN9*, the pCAMBIA1302-GmDHN9-GFP vector was constructed and expressed transiently in tobacco leaves. The results showed that *GmDHN9* was localized in the cytoplasm (Fig. 2).

### *Acquisition and Detection of Transgenic* Arabidopsis

**Figure 3.**
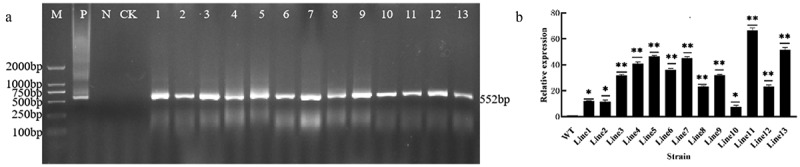
Detection of transgenic Arabidopsis. a: PCR detection of T_1_ generation transgenic Arabidopsis thaliana; b: fluorescence PCR detection of T_3_ generation transgenes. * *p* < .05; ** *p* < .01. All bars represent means ±SD, (*n* ≥ 3).

The recombinant plasmid pCAMBIA3301-GmDHN9 was transformed into wild-type Arabidopsis, and 13 positive lines of transgenic A. thaliana were obtained by Basta screening and PCR detection; the results are shown in Fig. 3a, while those of real-time PCR of T_3_ generation transgenes are shown in Fig. 3b. Three high-expression lines (Line5, Line11, Line13) were selected for follow-up experiments.

### *Overexpression of GmDhn9 Increased Germination Rate and Root Length in Transgenic* Arabidopsis

**Figure 4.**
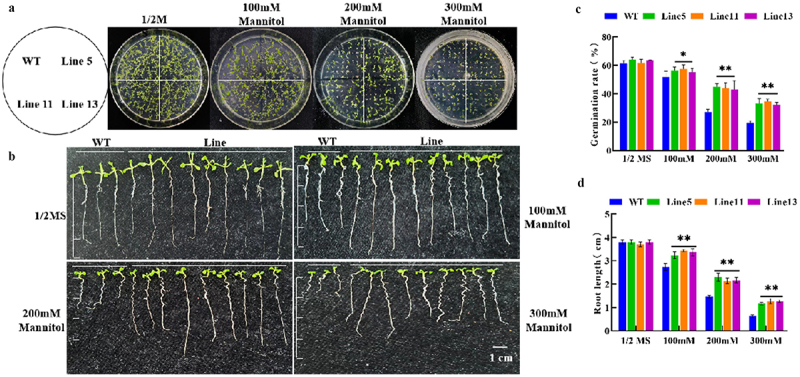
Determination of germination rate and root length of transgenic Arabidopsis thaliana. (a) germination rate phenotype; (b) root length phenotype; (c) germination rate statistics; (d) root length statistics. * *p* < .05; ** *p* < .01. All bars represent means ±SD, (*n* ≥ 3).

With the increase in mannitol concentration in the medium, the germination rate and root length of A. thaliana decreased. However, there were no significant differences in the germination rate and root length of A. thaliana between wild-type and transgenic lines on the media without mannitol. After adding mannitol, the germination rate of transgenic lines was significantly higher than that of wild-type A. thaliana (4-A, C) and root length, as shown in Fig. 4(b, d). The germination rate of the three transgenic plants was 5%, 18%, and 16% higher than that of the wild-type plants at three concentration gradients of 100 mM, 200 mM, and 300 mM, respectively, which indicated that the transgenic Arabidopsis thaliana possessed more robust drought tolerance.

### *Overexpression of GmDhn9 Improves Drought Stress Tolerance in Transgenic* Arabidopsis

**Figure 5.**
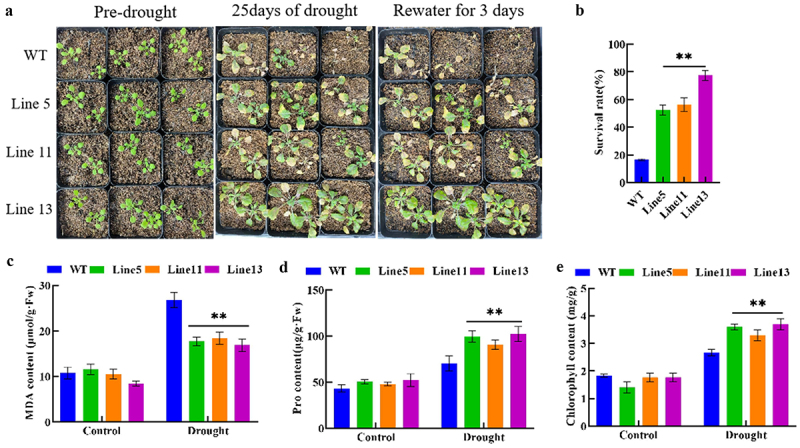
Phenotypic identification and determination of physiological and biochemical indexes of transgenic Arabidopsis thaliana. (a) rehydration experiment; (b) survival rate; (c) MDA content; (d) pro content; (e) chlorophyll content. * *p* < .05; ** *p* < .01. All bars represent means ±SD, (*n* ≥ 3).

To further validate the role of the *GmDHN9* gene in drought stress, we subjected transgenic Arabidopsis to natural drought stress for 25 days, followed by rehydration for three days, and determined the survival rate of transgenic Arabidopsis. We found that the survival rates of the three transgenic Arabidopsis plants under natural drought stress were 36%, 39%, and 61% higher than those of the wild-type plants, respectively. In addition, we determined the contents of chlorophyll, MDA, and Pro in plants under drought stress. Compared with wild-type A. thaliana, the MDA content (Fig. 5c) in transgenic plants was significantly reduced, while the contents of Pro (Fig. 5d) and chlorophyll (Fig. 5e) were significantly increased. These results showed that overexpression of *GmDHN9* improved the drought tolerance of transgenic A. thaliana.

### *Overexpression of* GmDhn9 *Reduces the Accumulation of ROS in Transgenic* Arabidopsis

**Figure 6.**
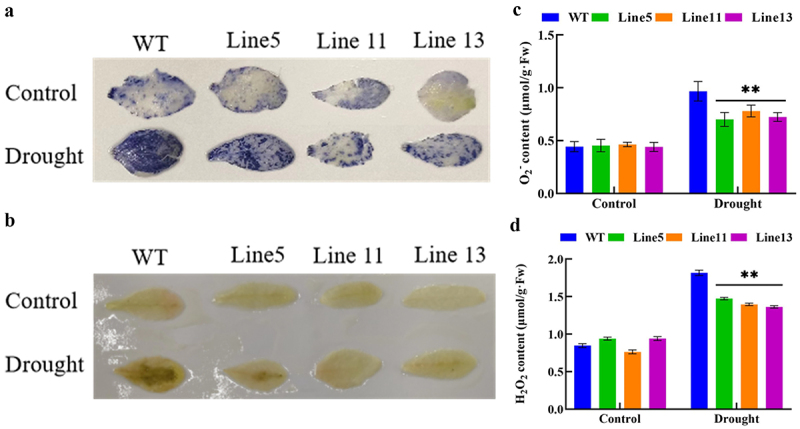
Detection of reactive oxygen species in transgenic Arabidopsis thaliana. (a) NBT staining; (b) DAB staining; (c) O_2_^−^ detection; (d) H_2_O_2_ detection. * *p* < .05; ** *p* < .01. All bars represent means ±SD, (*n* ≥ 3).

To explore whether *GmDHN9* can reduce ROS accumulation, we investigated ROS levels using NBT and DAB staining, and measured H_2_O_2_ and O_2_^−^ contents. The results are shown in Fig. 6(c, d). Under normal circumstances, there was no significant difference in H_2_O_2_ and O_2_^−^ contents between wild-type and transgenic A. thaliana lines. After drought stress, transgenic A. thaliana showed lower H_2_O_2_ and O_2_^−^ contents. The staining results using NBT and DAB (6-A, B) showed that the leaf color of wild-type A. thaliana was darker under drought stress, indicating that this plant accumulated more O_2_^−^ and H_2_O_2_, which were consistent with the detection results of O_2_^−^ and H_2_O_2_. This suggested that transgenic Arabidopsis lines had less ROS accumulation and cell membrane damage under drought stress. Therefore, it could be speculated that the expression of *GmDHN9* might improve the drought tolerance of plants by reducing ROS accumulation.

### *Overexpression of* GmDhn9 *Increases Antioxidant Enzyme Activity in Plants*

**Figure 7.**
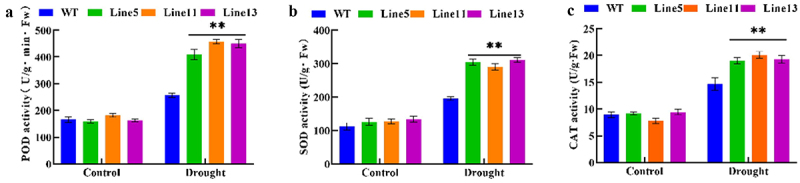
Detection of antioxidant enzyme activity in transgenic Arabidopsis thaliana. (a) POD activity; (b) SOD activity; (c) CAT activity. * *p* < .05; ** *p* < .01. All bars represent means ±SD, (*n* ≥ 3).

To detect the ability of plants to remove ROS, we tested the activities of SOD, POD, and CAT antioxidant enzymes. Compared with wild-type A. thaliana under drought stress, the activities of SOD, POD, and CAT in transgenic plants were significantly improved. These results are shown in Fig. 7, indicating that transgenic A. thaliana had a stronger ability to clear ROS and an increased tolerance to drought stress. Therefore, it could be speculated that the expression of *GmDHN9* might improve the drought resistance of plants by increasing the activity of antioxidant enzymes, thereby reducing ROS accumulation.

### Transgenic A. thaliana Positively Regulates the Expression of Drought-Related Genes

**Figure 8.**
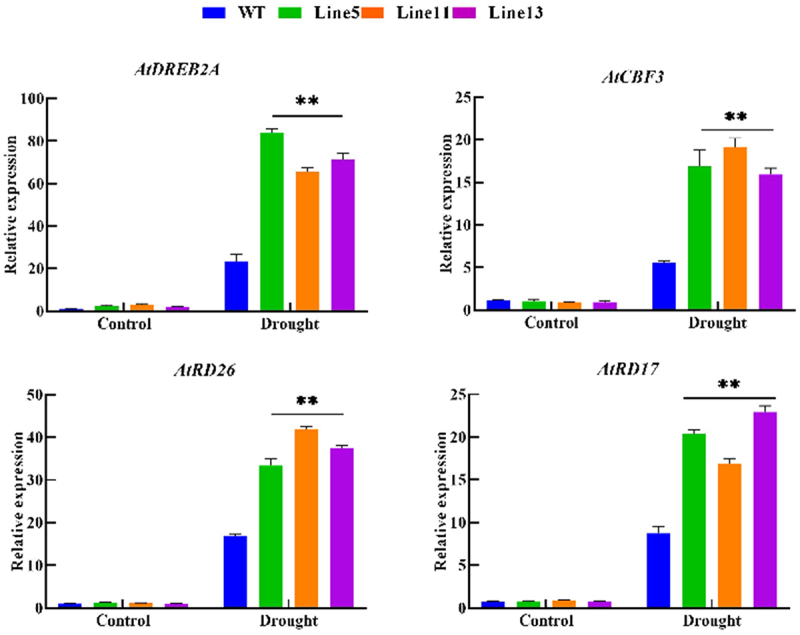
Detection of drought-resistant gene expression in T_3_ generation transgenic Arabidopsis thaliana. * *p* < .05; ** *p* < .01. All bars represent means ±SD, (*n* ≥ 3).

The expression level of drought-resistant genes can be used to assess the response-ability of plants to drought stress, and related genes such as *DREB2A*, *RD17*, *RD26*, and *CBF3* were selected since they significantly upregulate expression under drought stress. This experiment examined the relative expression levels of these four genes in wild-type and transgenic A. thaliana. Through the analysis of qRT-PCR results, the expression levels of genes related to *DREB2A*, *RD17*, *RD26*, and *CBF3* were significantly higher than those of wild-type plants under drought stress, as shown in Fig. 8. Therefore, *GmDHN9* may regulate the genes associated with plant drought stress, thereby positively regulating the response of plants to drought stress.

## Discussion

Highly hydrophilic DHN protein acts as a protective agent against drying and accumulation in the embryo.^25^ When bound to ligands (e.g., membranes), it can hinder the aggregation of other molecules, replacing inactivated chaperone proteins in the dry state to maintain the biological activity of cells.^33^ Studies have reported that the expression of *DHN* genes plays an important role in coping with abiotic stresses such as plant drought, salinity, and low temperature.^34,35^ The results of this study were analyzed using the promoter sequence of the gene and it was seen that the *GmDHN9* gene had related cis-acting elements in response to drought, dehydration, abscisic acid, and ABRE. Wild-type soybeans were treated with 10% PEG6000, 100 μM ABA, 100 mM NaCl, and low temperature (4°C) for 0, 3, 6, and 12 h. The qPCR results showed that *GmDHN9* responded to drought, salt, ABA, and low temperature of 4°C, and the response to drought stress was stronger. Subsequently, subcellular localization of *GmDHN9* was performed to verify its localization in the cytoplasm.

Under drought stress, many plants protect and stabilize subcellular structures by accumulating proline, buffering cellular redox potential, maintaining photosynthesis, scavenging free radicals, and regulating cell function.^36^ The content of plant MDA can reflect the degree of damage to plant cell membranes. *HbDHN1* and *HbDHN2* enhanced the Pro content, which in turn improved the plant tolerance of bananas to drought and osmotic stress.^37^ Maize *ZmDHN13* enhanced the tolerance of transgenic tobacco to oxidative stress.^38^ The *EjDHN1* gene enhances cold tolerance in transgenic tobacco by mitigating oxidative responses and protecting against cell membrane damage.^39^ In this study, compared with wild-type A. thaliana after drought stress treatment, the germination rate, root length, chlorophyll content, proline content, relative water content, and survival rate of transgenic ones were significantly increased, and the content of MDA was significantly reduced. It was suggested that overexpression of *GmDHN9* enhanced the drought resistance of A. thaliana.

**Figure 9.**
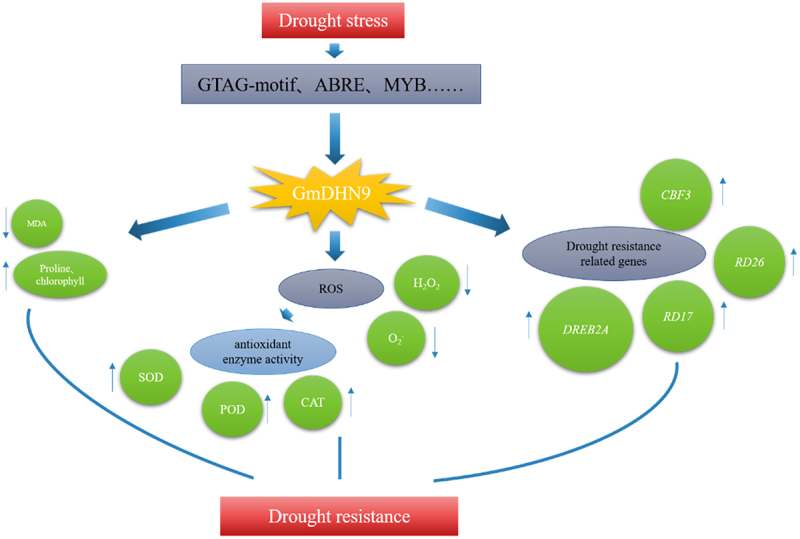
*GmDHN9* gene mediates drought tolerance mechanism in Arabidopsis thaliana.

Plants have an antioxidant defense system that can effectively remove the accumulation of ROS in plants and can maintain ROS balance and intracellular redox homeostasis.^40^ Drought peroxidizes cell membranes, and antioxidant enzymes eliminate superoxide radicals to mitigate membrane lipid peroxidation and membrane structure damage.^41,42^ It was seen that the drought stress tolerance of rice improved through excessive accumulation of antioxidant flavonoids.^43^ In Arabidopsis, KS-type dehydrin *AtHIRD11* inhibited the production of H_2_O_2_ and hydroxyl radicals in the copper ascorbate system to improve drought resistance.^44^ In this study, the contents of H_2_O_2_ and O_2_^−^ in transgenic A. thaliana were significantly reduced, and the activities of POD, SOD, and CAT were significantly increased after drought stress treatment, indicating that less ROS accumulated in them, and their ability to remove ROS was stronger, and it had greater tolerance to drought stress. We performed qRT-PCR on genes involved in drought stress (*DREB2A*, *RD17*, *RD26*, and *CBF2*). The results showed that the expression of all genes was up-regulated under drought stress, and the drought tolerance mechanism mediated by the *GmDHN9* gene in Arabidopsis is shown in Fig. 9. In summary, *GmDHN9* may be a positive regulator of drought tolerance in plants.

## Conclusion

The present study determined that *GmDHN9* was a positive regulator of drought tolerance in Arabidopsis, and subcellular localization results showed its localization in the cytoplasm. Through phenotypic, physiological, biochemical, and molecular experiments on transgenic A. thaliana under drought stress, it was shown that overexpression of *GmDHN9* increased their root length, germination rate, chlorophyll and Pro contents, reduced the contents of MDA, H_2_O_2_, and O_2_^−^, thereby improving the drought tolerance of plants. Furthermore, it enhanced the tolerance of these plants to drought stress by increasing antioxidant enzyme activity and removing accumulated ROS.

## Supplementary Material

Supplementary clean.docx
