# Supplementary material for: Overexpression of soybean GmDHN9 gene enhances drought resistance of transgenic Arabidopsis
Source: GM Crops Food. 2024 Apr 2;15(1):118–29. doi: 10.1080/21645698.2024.2327116 (PMC10989702; doi:10.1080/21645698.2024.2327116)
Supplement: Supplementary clean.docx [file KGMC_A_2327116_SM1575.docx]

Table S1. Cis-elements in the promoter region (~2 kb) of GmDHN9

| abbreviation | quantity | function |
| --- | --- | --- |
| ABRE | 1 | cis-acting elements involved in abscisic acid reactions |
| ARE | 1 | Cis action regulatory element necessary for anaerobic induction |
| AT-rich element | 1 | Binding site of AT-rich DNA-binding protein (ATBP-1) |
| AT~TATA-box | 7 | Core promoter element |
| Box 4 | 1 | Part of the conserved DNA module involved in photoreactions |
| Box II | 1 | Part of a photoresponsive element |
| CAAT-box Conservative sequence | 40 | The promoter and enhancer regions share cis-acting elements |
| CAT-box | 1 | Meristem expresses regulatory elements |
| CTAG-motif | 1 | Abscisic acid response element |
| ERE | 1 | Ethylene response element |
| G-Box | 1 | A cis-action regulatory element involved in the light response |
| GARE-motif | 1 | Gibberellin response element |
| GT1-motif | 1 | Light response element |
| HD-Zip 1 | 1 | Elements involved in the differentiation of fence mesophyll cells |
| LAMP-element | 1 | Light response element |
| MBSI | 1 | MYB synthesis sites involved in regulating flavonoid synthesis |
| MRE | 1 | Myb binding sites involved in photoreactions |
| MYB | 7 | Responding to drought high salt low temperature cis elements |
| MYB-like sequence | 2 | Responding to drought high salt low temperature cis elements |
| MYC | 6 | Dehydration response element |
| P-box | 1 | Gibberellin response element |
| RY-element | 1 | Cis regulatory elements in seed-specific regulation |
| TATA | 1 | Core promoter element |
| TATA-box | 47 | Core promoter element near transcription start site-30 |
| TCA-element | 1 | cis-acting element involved in the salicylic acid reaction |
| TCCC-motif | 1 | Partial light response element |
| Unnamed__1 | 1 | 60k protein binding site |
| W box | 1 | WRKY binding site, associated with salicylic acid |
| WUN-motif | 1 | Damage-inducing action element |

Table S2. Primer information in this study.

| Primer name | Primer sequences（5’—3’） |
| --- | --- |
| *115*F | TTGAGAAGCAAACCGACGAA |
| *115*R | GCAGTTCTGGTGGCTACATAGGT |
| *GmActin*-F | GCGGGAAATTGTAAGGGATGT |
| *GmActin*-R | TCGCCAATAGTGATGACCTG |
| *115xin*-UP | GGGCCATGGTAGCAAGTTATCAAAAGCAC |
| *115xin*-LOW | GGTCACCCTACTTGTCACTGTGTCCTC |
| *Bar* S | AAACCCACGTCATGCCAGTT |
| *Bar* AS | GTCTGCACCATCGTCAACCAC |
| *AtEF1*-F | AGAAGGGTGCCAAATGATGAG |
| *AtEF1*-R | GGAGGGAGAGAGAAAGTCACAGA |
| *AtDREB2A*-F | TGACCTAAATGGCGACGATGT |
| *AtDREB2A*-R | TCCAAGTAACTCAAGTCGTCG |
| AtCBF3-F | GATGACGACGTATCGTTATGGA |
| *AtCBF3*-R | TACACTCGTTTCTCAGTTTTACAA |
| *AtRD17*-F | ACGTCCACGCCGTTGGT |
| *AtRD17*-R | CTCCGGATGTTCCACTGGAA |
| *AtRD26*-F | GAAGGTGAGGCGGAGAGTG |
| *AtRD26*-R | CCCGAAACTCTGAGTCAACCT |


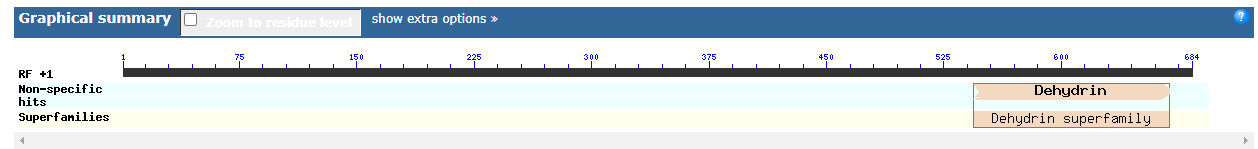


Figure S1. Analysis of conserved structural domains of the *GmDHN9* gene
